# Supplementary material for: Patterns of Intron Gain and Loss in Fungi
Source: PLoS Biol. 2004 Nov 30;2(12):e422. doi: 10.1371/journal.pbio.0020422 (PMC532390; doi:10.1371/journal.pbio.0020422)
Supplement: Table S1 — Also available at http://genes.mit.edu/NielsenEtAl/. (4.3 MB ZIP). [file pbio.0020422.st001.zip › NielsenEtAl/html/561.html]

AN0653.1.NCU00303.1.MG00364.1.FG05533.1


```
 CLUSTAL W (1.82) Multiple Sequence Alignments - Introns Inserted


Sequence 1: NCU00303.1	255 aa
Sequence 2: MG00364.1	241 aa
Sequence 3: AN0653.1	236 aa
Sequence 4: FG05533.1	231 aa
Alignment Length: 260 aa
Number Identitical Residues: 84 aa
Alignment Score (without introns) 4705


MG00364.1 	-MSIPMTRLGDR-~---------E1AEYDRLRDLARQEARKRGDCFER0SRQAYENGDGA
NCU00303.1	-MAIPMERLGDRA1FNHRTSVEIE~SEYDRLRAEARAEGDKMKRYFDE0AHQAYERGDGA
FG05533.1 	-MSIPMQRLGGRA1FAHDDD-SIE~QEYDRLRDMARAEAEKRNDCFAR0SRQAYEDGDGA
AN0653.1  	MHEHELSYMGPRA1FNHSQSNDAE~SEYDRLRDLARQEASKRNSCFQR0SQEAYASGDGA
          	     :  :* *:      . . *  ******  ** *. *    * . :::**  ****

MG00364.1 	EAKRLSNEGKKHGANMERYNKQASEYIFRENNNPERVAEDTIDLHGQFVEEAEDILEARI
NCU00303.1	EAKNLSNLGKKHKAKRDAINKQAAEFIFRENNHPERVAADTIDLHGLYVEEAEDILEARI
FG05533.1 	GAKELSNQGKAHAARMDDYNQQASDFIFRENNASGRVEADSIDLHGLYVEEAERILEERI
AN0653.1  	AAKELSEQGKAHGRKMEEYNRQASEFIFRENNAPGRVDADTIDLHGQFVEEAEDILEERI
          	 **.**: ** *  . :  *:**:::****** . **  *:***** :***** *** **

MG00364.1 	RDAQARGQRHLHV~IVGKGNHSAGGVQKIKPRVEQVCRELGLDYATEA-NDGRIFVDLTG
NCU00303.1	RDAQARGQTHLHV~IVGKGNHSAGGVRKIAPRVEQVCREMGLNFAAEE-NEGRIYVDLTG
FG05533.1 	RSDQAKGQTHLYA~IVGKGHHSAGGVQKLKPKVEELCQELGLRYETDEDNTGRIIINLQG
AN0653.1  	KYARSQGQTHLHV2IVGKGNHSANHVQKIKPRVEKVCRELGLQYATEE-NEGRIYVNLTG
          	:  :::** **:. *****:***. *:*: *:**::*:*:** : ::  * *** ::* *

MG00364.1 	AKVGAPPPLPPQPGGGYGGGGHGPSHGGHQPHHDSGYGQAHHGGGQYQQHPPQQQQQHHQ
NCU00303.1	GRVDGLPGLPQQPPGYQPGGGYQ-SEG--RPNQHHGGGGGGYPGQQQQQHGQQNHQQGQQ
FG05533.1 	GQ----PVPP--------------SHS---------GGHGGYPGGEQHHGGPHHGGQHHQ
AN0653.1  	GAADSLPSKP--------------SHG-------HGHQQQHY--QQPHQHQQQQQGNQDE
          	.  .. *  *              *..        .     :   : ::   ::  : .:

MG00364.1 	QQQQQQD--DDIIG1ALRWA-----
NCU00303.1	QNQQEDDIWADLLG~ACFKKCCVVM
FG05533.1 	PEEKNDDLVAIIFK~KLEKMCCTVM
AN0653.1  	IEQVVNAILPRVLR~KLEKACCVVM
          	 ::  :     ::       ..
```
